# Non-Milk Extrinsic Sugars Intake and Food and Nutrient Consumption Patterns among Adolescents in the UK National Diet and Nutrition Survey, Years 2008–16

**DOI:** 10.3390/nu11071621

**Published:** 2019-07-17

**Authors:** Heidi T. Lai, Jayne Hutchinson, Charlotte E. L. Evans

**Affiliations:** 1 Nutritional Epidemiology Group (NEG), School of Food Science and Nutrition, University of Leeds, Leeds LS2 9JT, UK; 2 Friedman School of Nutrition Science and Policy, Tufts University, 150 Harrison Ave, Boston, MA 02111, USA

**Keywords:** free sugars, added sugars, non-milk extrinsic sugars, diet quality, nutrient intake

## Abstract

The revised guidelines from the Department of Health (DoH) in the UK state that mean population intakes of free sugars should be below 5% of the total energy (TE) consumption of the British population. However, very few studies have assessed the impact of this recommendation on diet quality in the UK. We explored the dietary patterns and intakes of micronutrients of British adolescents with low intakes of non-milk extrinsic sugars (NMES) (similar to free sugars but not equal, with slight differences in the categorisation of fruit sugars from dried, stewed or canned fruit and smoothies), using the National Diet and Nutrition Survey Rolling Programme, years 1–8 (NDNS RP). The sample included 2587 adolescents aged 11–18 years. Four percent (112) of adolescents reported consuming 5% or lower NMES as a proportion of TE. The odds of being categorised as a low-sugar consumer in adolescents (≤5% TE from NMES) were significantly lower with higher intakes of sweetened drinks, fruit juice, cakes, biscuits, sugar and sweet spreads, chocolate confectionery and sugar confectionery, and significantly higher with higher intakes of pasta and rice, wholemeal and brown bread, and fish. Across the five categories of NMES intakes, micronutrient intakes were lowest for those consuming either ≤5% TE or more than 20% TE from NMES, and optimal for those consuming between 10–15% of energy from NMES. These findings confirm the difficulties of meeting the free sugars recommended intake for adolescents. Care needs to be taken to ensure that an adequate consumption of micronutrients is achieved in those adhering to the revised guidelines on free sugars.

## 1. Introduction

The prevalence of obesity is high in the UK [1,2]; nearly 25% of adults are obese and the risk of obesity in adulthood is much higher for those who are obese in childhood or adolescence [3]. The causal factors for obesity are complex and multi-factorial, but many are modifiable through individual and policy action to improve dietary and activity behaviour. As such, the World Health Organization (WHO) recommends that individuals reduce their intakes of fats and sugars and increase their consumption of fruits and vegetables to improve their health [2], which includes limiting the consumption of free sugars in foods and drinks.

There are several factors which suggest that a diet high in non-milk extrinsic sugars (NMES) could result in a poor-quality diet, including excess energy intake, low satiety, poor compensation in terms of energy intake, a less nutritious diet higher in nutrient-poor foods and lower in nutrient-rich foods. NMES are similar to free sugars [4] and was the definition used for recommending sugars intakes before 2015 in the UK. Ultimately, a diet rich in these sugars results in weight gain [5,6]. Based on further evidence from systematic reviews of dietary sugars and body weight [7], and on dental caries [2], the WHO and the Scientific Advisory Committee on Nutrition (SACN) revised the recommendations to restrict added and free sugars intake in 2015. The recommended % total energy (%TE) from free sugars was lowered from 10% TE [8] to 5% TE [9].

To date, no national studies have reported on diet patterns with different categories of NMES intake. Two studies assessed micronutrient adequacy by dietary sugar intake [10,11], but reported no firm basis to describe an optimal intake of added sugars with regard to micronutrient adequacy, given how divergent the reported relationships were between micronutrients and added sugar were across studies. Whilst most studies report either no association between added sugars intakes and dietary adequacy or some deterioration with high intakes, some also describe a curvilinear association with poorer micronutrient status at the lower extremes of added sugars intake [12]. This may be related to low overall food intakes in low-sugars consumers, which could be due to deliberate energy restriction for weight reduction, distorted reporting of all or specific foods, or avoidance of foods which are particularly rich sources of micronutrients.

Whilst it is clearly important to determine the impact of high consumption of free or added sugars, it is equally important to explore associations with micronutrient intakes in individuals adhering to the guidelines on added or free sugars, as significant deviations from the general UK dietary pattern might have been adopted [13]. Our study therefore aimed to examine the potential impact of adherence to the revised guidelines on the intakes of important key foods and nutrients in British adolescents. We quantified existing dietary intakes of major food groups and nutrients in participants of the National Diet and Nutrition Survey Rolling Programme years 1–8, categorised by percentage of energy from NMES.

## 2. Materials and Methods

### 2.1. The National Diet and Nutrition Survey Rolling Programme

In the UK, the National Diet and Nutrition Survey Rolling Programme (NDNS RP) provides an authoritative source of information on the nutritional status of the UK population, providing descriptors of food and nutrient intakes, biomarkers of nutritional status and anthropometric indices of over, and underweight. This survey is funded by the Public Health arm of the Department of Health for England (Public Health England) as a means of monitoring diet and nutrient trends and the adequacy of the UK diet. Fieldwork was carried out between 2008 and 2016 to collect dietary and lifestyle information from approximately 1000 participants every year from private households, providing sufficient statistical power to observe differences between dietary intake groups. Further details of the survey and sampling methods were reported elsewhere [14]. Data files from years 1–8 of the NDNS Rolling Programme (2008–2016) were obtained under licence from the UK Data Service.

### 2.2. Dietary Information

Dietary information was collected using a four-day food diary. The participants were required to complete the diary by reporting portions of food and drink consumed, using household measures over four consecutive days assigned randomly by the interviewer’s computer-assisted personal interview (CAPI), beginning on any day of the week. For children aged 11 years, a parent/carer was asked to complete the four-day diary with help from the child as appropriate. Photographs of food portions were included in the diary to aid portion size descriptions and younger children (<16 years) were provided with an age-appropriate version of the Young Person’s Food Photograph Atlas [14]. Consumption was expressed as grams per day (g/day). Data on food group consumption and total nutrient intakes averaged over 4 days (and in some cases 3 days) were provided for each adolescent participant.

### 2.3. Characteristics

Participant characteristics were collected using a CAPI programme and self-completion questionnaires during the interviewer visit in the first stage of the survey. Classification of socio-economic status was undertaken based on occupation, according to the UK National Statistics-Socio-Economic Classification (NS-SEC). Participants were initially divided into eight NS-SEC categories and reclassified into three categories: (1) managerial/professional, (2) intermediate, and (3) routine/manual, in addition to ‘unemployed/don’t know’ or ‘missing’. Height and weight were measured, and the mean of three valid measurements were recorded, from which BMI (kg/m^2^) was derived. Waist circumference (cm) and waist-to-hip ratio measurements were taken during a consented nurse visit during the second stage of the survey. Waist and hip circumference (cm) was measured, and the mean of three valid measurements were recorded.

### 2.4. Statistical Analyses

Survey weights from the dataset were applied to account for bias in non-response and probability of selection by age, sex and Government Office Region relative to the total population in the UK, as addressed elsewhere [14,15,16].

The food groups (Supplementary Table S1) investigated here are similar to previous literature, and a full list can be found in the supplementary tables in the NDNS report [15]. In this study, we focused on foods high in sugar, as well as foods which provide alternative substantial energy from protein, fat, and carbohydrates. Sugar sweetened drinks included carbonated and cordial drinks but not pure fruit juice, milk-based drinks or tea and coffee. A number of different dietary sugar variables were derived from the food diary analysis. In line with the SACN recommendation, NMES as a percent of total energy was the variable used for this analysis (rather than percent of food energy). However, the proposed SACN guidelines refer to ‘free sugars’ which also include sugars in pure fruit juice and 50% of sugars in fruit purees that are not included in NMES; therefore NMES values are likely to be slightly lower than free sugars levels [13]. Participants were categorised by percentage (%) of total energy from NMES into 5 groups (≤5%, >5–10%, >10–15%, >15–20%, >20%) with means reported. Wald tests were carried out to determine statistically significant differences in mean characteristics, such as anthropometric measures, between the NMES consumption categories. Chi-square tests were used to determine differences in categorical variables, such as smoking, between NMES consumption groups reported as percentages and 95% confident intervals (CI) in table 1.

Energy, food and nutrient intakes (excluding supplements) by the %NMES category were reported as means (g/day and mg/day or µg/day) and 99% confidence intervals (CI). Wald tests were carried out to determine statistically significant differences in intake between the % NMES categories. Patterns of food consumption were visualised using a radar chart. Logistic regression was undertaken to determine the odds (99% CI) of being classified as a low NMES consumer (≤5% NMES) compared with any other NMES category with increasing food intake by typical portions (g/day). This was adjusted by age and gender. Sensitivity analyses excluded 6.5% of participants, who were dieting (*n* = 152). We also adjusted for those who reported dieting. When dieters were excluded, only 94 individuals remained in the lowest NMES group for the sensitivity analyses. The proportion of adolescents consuming less than the Lower Reference Nutrient Intake (LRNI) for vitamins A, C, B12, riboflavin, and folate, and the minerals iron, calcium, magnesium, potassium, zinc and iodine was reported as a % and 99% CI by category of %NMES consumption, and a graph of the % was produced. Stata version 14.1 was used for all statistical analyses and statistical significance was determined using *p* ≤ 0.01 due to multiple testing.

The extent of under-reporting was explored using estimates of the basal metabolic rate derived from standard Harris–Benedict equations [17] multiplied by a very low physical activity level (PAL) of 1.2 [18]. This value was used to reflect an implausible level of energy intake reported from the 4-day survey diary. However, under-reporting using even this conservative approach was so pervasive and generated such high numbers of potential under-reporters that their exclusion would render the analysis unfeasible. Accordingly, no individuals were excluded on the basis of under-reporting.

### 2.5. Research Ethics

Ethical approval for the NDNS RP had already been obtained from the Oxfordshire A Research Ethics Committee. The letters of approval for the original submission and subsequent substantial amendments, together with the approved documents, were sent to all the Local Research Ethics Committees (LRECs) covering the areas where the fieldwork was conducted. No further approval was required.

## 3. Results

The analysis was carried out on 2587 adolescents aged 11 to 18 years. Their mean intake of NMES in grams was 72 g/day (95% CI 70 to 74), and as a percentage of total energy intake, this was 14.9% (95% CI 14.5 to 15.2). They were categorised by level of % total energy from NMES consumption, as described in the methods, and 4% (*n* = 112) of the sample consumed ≤5% NMES, and therefore met the recommended level of intake. This category consumed a mean of 13 g of NMES. The category with the highest number of participants (34%) consumed 10–15% of energy from NMES with an average daily intake of 60 g/day of NMES. The highest NMES consumption group, with intakes greater than 20% of total energy, included almost a fifth of the sample (18%), with an average daily intake of 122 g/day of NMES.

Few statistically significant differences in participant characteristics between the categories of NMES consumption were observed (see Table 1) but individuals from ethnic minorities were more likely to be in the lowest NMES consumption group. Although more females and obese individuals tended to be in this group, differences were not statistically significant. The proportion of adolescent participants within each NMES intake category with implausible recorded energy intakes was consistently high across all categories, but was markedly greater in the lowest NMES consumers at 79%, compared with 49% in the highest consumers.

### 3.1. Foods

Differences in mean intake (g/day) of selected foods by category of sugars consumption (% energy) are displayed in Table 2. There was a general trend across the categories for pasta and rice, wholemeal bread and high fibre breakfast cereals to be eaten in larger quantities in the lower NMES categories. Conversely, consumption of biscuits, cakes, and ice-cream was higher with each increase in the NMES category; however, no significant difference was observed for pudding intake. Confectionery, sugars and sweet spreads increased with increasing added sugars across the categories. Sweetened soft drinks, fruit juices and beer consistently increased over all the NMES categories, but low-calorie drinks did not show a clear trend across the categories. Non-low-calorie soft drinks consumption was particularly high in the highest NMES category, with a daily mean intake of about 500mls in the highest category compared with 12 mL in the lowest. The highest intakes of cheese, yogurt and other dairy desserts were found in the middle groups of NMES intake. The intake of savoury snacks, such as crisps, increased over increasing sugars categories. The intake of disaggregated total fish and vegetables generally decreased across increasing sugars categories.

**Figure 1:**
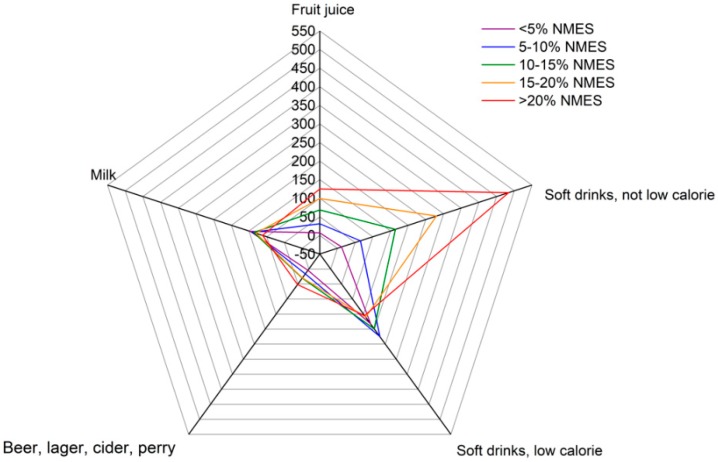
Consumption of alcoholic and non-alcoholic beverages (g/day) by adolescents in the National Diet and Nutrition Survey aged 11 to 18 years, by category of non-extrinsic milk sugar after the application of survey weights.

**Figure 2:**
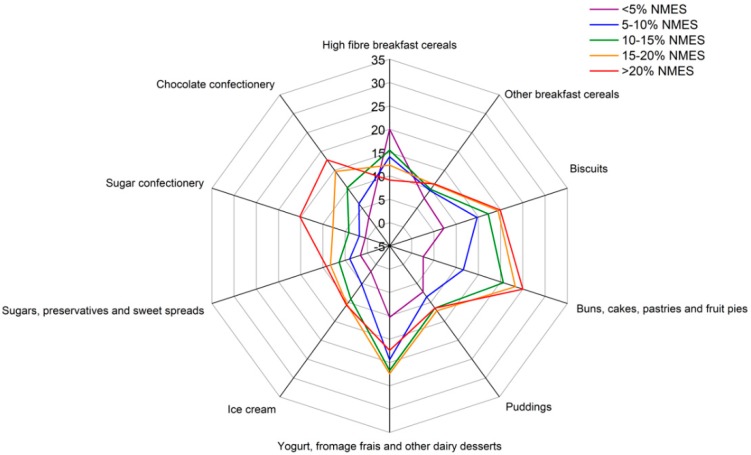
Consumption of sweet foods (g/day) from adolescents aged 11 to 18 years in the National Diet and Nutrition Survey, by category of non-extrinsic milk sugar after the application of survey weights.

Differences by category of NMES for the selected drinks and foods are also displayed in radial graphs for ease of interpretation (Figure 1 and Figure 2, respectively). Participants in the higher categories of NMES consumption had high intakes of full-sugar soft drinks, which was highest in the highest NMES category and lowest in the lowest NMES category. The remaining drinks such as milk, fruit juice and low-calorie soft drinks varied little by NMES category. The participants in the higher NMES categories had particularly high intakes of biscuits, cakes and both sugar and chocolate confectionery. There was less variation between the categories of NMES for puddings, yogurt and other dairy desserts, breakfast cereals, ice-cream and sugars and sweet spreads. Mean intakes by weight were highest for cakes, chocolate confectionery, and yogurts.

**Figure 3:**
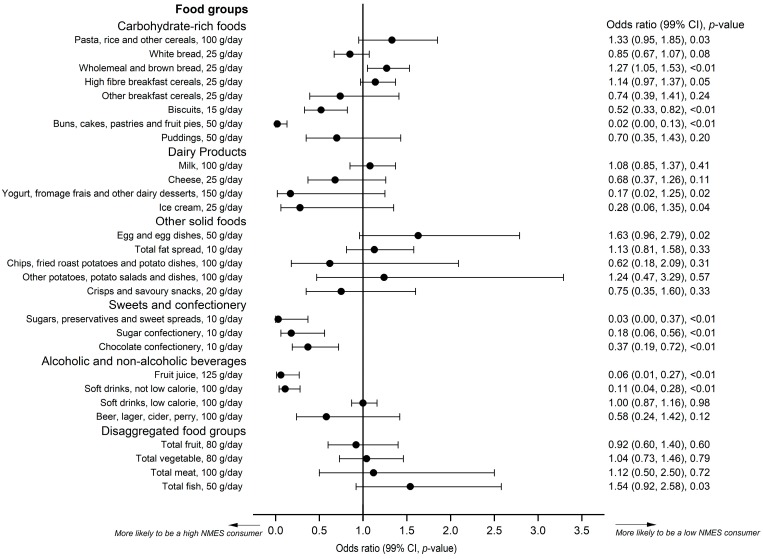
The odds (99% CI, *p*-value) of adolescents aged 11 to 18 years being categorised as consuming <10% NMES of total energy with increasing consumption of various foods by portion (g/day) in the National Diet and Nutrition Survey.

The odds of being categorised as a low-sugars consumer (≤5% NMES) varied by food type. The age- and gender-adjusted results are provided in Figure 3. The odds of an adolescent being categorised as a low-sugars consumer compared with any of the other NMES categories were significantly lower with greater consumption of biscuits, cakes, sugar and sweet spreads, confectionery, fruit juice and full-sugar soft drinks. The odds of being categorised as a low-sugars consumer were significantly higher with higher intakes of wholemeal and brown bread. Similar findings were reported when dieters were excluded or adjusted for, although on exclusion of dieters, the odds were also significantly lower in relation to greater consumption of ice-cream and significantly higher with greater consumption of eggs, but not significant for wholemeal bread (see Supplementary Table S2).

### 3.2. Nutrients

Energy and nutrient intakes by category of percentage of energy from NMES are reported in Table 3. Energy intakes (both food-derived and total) were consistently lowest in participants reporting the smallest intakes of NMES, increasing with increasing NMES intake. Protein and dietary fibre were lowest in the highest NMES group, and fibre was consistently low across all the NMES intake categories. Intakes of energy from total fat and total carbohydrate were reciprocally associated with higher carbohydrate and lower fat intakes in the highest NMES consumers. Intakes of alcohol were low, as might be expected, but were equivalent to about 2 units per week on average in the highest NMES consumers.

In terms of water-soluble vitamins, intakes of riboflavin, niacin equivalents and B12 tended not to vary greatly by NMES intake category. However, the highest folate, zinc, magnesium, calcium, iron, vitamin E, Vitamin D, iodine, potassium and sodium intakes were consumed in the middle NMES consumer group (>10–15%), whilst vitamin C intakes increased with increasing NMES consumption. All micronutrient intakes were lower in the lowest NMES intake group (meeting recommended NMES levels) compared with those in the intermediate categories (5–10% and 10–15% of energy categories). The results were similar, albeit slightly attenuated when dieters were excluded (see Table S3 and Figure S1).

**Figure 4:**
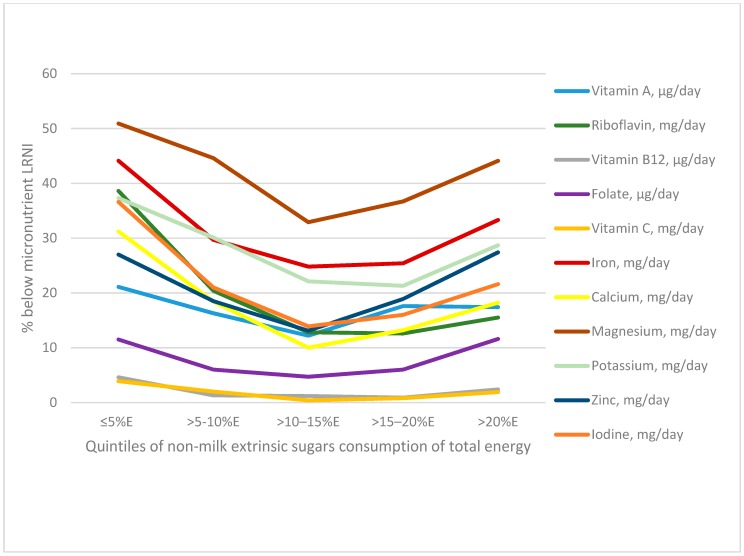
Percentage of adolescents aged 11 to 18 years in the National Diet and Nutrition Survey with micronutrient intakes below LRNI by percentage of non-milk extrinsic sugars consumption of total energy (*n* = 2587) after the application of survey weights.

Table 4 shows the percentage of adolescents consuming less that the LRNI (very low micronutrient consumers who are likely to be deficient if usual intake is below this level) for micronutrients by category of percentage energy from NMES. Generally, for the nutrients of concern, there was evidence of a U-shaped relationship between the percentage of energy from NMES and the proportion of each category reporting less than the LRNI for vitamins A, riboflavin and folate and also for the minerals calcium iron, magnesium, potassium, zinc and iodine (see Figure 4). Those consuming between 10–15% of energy from NMES had the smallest proportion of individuals consuming below the LRNI for most micronutrients. The lowest and highest NMES consumer categories had the greatest percentage of individuals consuming less than the LRNI. For example, 44% in the lowest NMES intake category, and 33% in the highest category did not consume more than the LRNI for iron, compared with 25% to 30% of participants in the middle NMES categories. 20% or more of participants with the lowest NMES intakes reported consuming less than the LRNI for vitamin A, riboflavin, iron, calcium, magnesium, potassium, zinc and iodine.

## 4. Discussion

The intakes of NMES in UK adolescents within this nationally representative UK survey were 14.9%TE (75 g/day), with only 4% meeting the current UK or WHO [2] recommendations currently set at ≤5%TE. Twenty-one percent consumed less than 10%TE from NMES, and were therefore adherent to the previous UK recommendations set by the Department of Health in 1991 [8]. The low-sugars consumers consumed less sugar sweetened drinks, fruit juice, biscuits, cakes, sugar and sweet spreads, confectionery, yoghurts and ice-cream. Furthermore, low-sugars consumers ate more vegetables, pasta and rice, wholemeal and brown bread, and fish. In terms of nutrients, the NMES intake category with the lowest proportion of adolescents that were deficient (intakes below LRNI) was the 10–15% NMES category.

Our findings for average NMES intake among adolescents are similar to the NHANES (National Health and Nutrition Examination Survey) in the US (both surveys report approximately 14%TE for all age groups) [19]. Adolescents who meet the ≤5% energy from NMES recommendation were similar in numbers to those observed in other European countries, and consistent with observations elsewhere of higher consumption levels in adolescents than in adults [20]. In a recent analysis of the Dutch National Food Consumption Survey 2007–2010 [21], adherence to the ≤ 5% recommendation was even lower, at < 1% of the sample, suggesting that an NMES intake ≤5% TE might be too low to achieve within the general UK population and other countries. Interestingly, adolescents who consume 10–15% NMES were the least likely to be deficient in many micronutrients, and thus, arguably, consumed the most nutritionally balanced diets. It is likely that adolescents with extremely low NMES intakes are consuming diets atypical to the general population due to restrictions in intake, including dieting, as they also had much lower energy intakes than the remaining categories. As such, substantial changes in dietary patterns are needed to adhere to the new recommendations, not simply removing high-sugar foods from the diet.

Given this potential major shift in nutritional intake for the majority of the UK population, in order to achieve compliance, it is therefore important to have confidence that there is no evidence of detriment in terms of dietary micronutrient adequacy. However, our findings suggest a U-shaped relationship between odds of micronutrient inadequacy and NMES intake by %TE for several micronutrients, where adolescents with the lowest %TE from NMES appeared to have the highest likelihood of micronutrient inadequacy. This is broadly consistent with a systematic review of 30 cross-sectional and prospective studies investigating associations between added sugar and nutrient intakes, where 21 found a negative association between added sugar and micronutrient intakes [22]. Our finding that micronutrient inadequacy increased with higher NMES consumption was consistent with the systematic review. However, it is unclear whether the observed effects from the systematic review are clinically meaningful, as the differences were reported to be small to moderate. None of the previous studies in the review reported a positive association with lower diet quality at lower sugar intakes, as we saw here. This may be because none of the studies in the review specifically compared categories of sugars at 5% or below with higher intake categories; 10% of sugars intake or below was the lowest category reported by any of the included studies. No mention was made of excluding dieters in the review but dieters or participants restricting their diet in other ways could be dominating this low-sugars group. However, we did not see any major differences in dietary patterns or nutrient adequacy in the low-sugars consumers when dieters were excluded.

Among food categories, drinks consumption, especially high-calorie soft drinks, is perhaps the strongest driver of added sugars intake, followed by confectionery, cakes and biscuits. These food groups were strongly associated with the odds of a 10-fold increase in NMES intake (13 g vs. 122 g) between the lowest and highest sugars consumers. This is consistent with earlier findings by a repeated cross-sectional study of 1991 English school children aged 11–12 years that reported substantial increases in the percentage of added sugars from drinks and breakfast cereals since 1980 [23], and that sugar sweetened drinks are the highest source of added sugars in most age groups [14]. In contrast, we did not find significantly higher intakes of breakfast cereals, either whole-fibre or other cereals in high-sugars consumers in comparison to the repeated cross-sectional English study [23], perhaps because sugars from breakfast cereals contribute a relatively small amount to the overall intake of NMES despite increased breakfast cereal consumption in the last two decades.

We also found a clear relationship between %TE from NMES and total energy intakes, with participants in the highest NMES intake category reporting intakes that provided 35% more energy than the lowest consumers. This supports contentions that higher intakes of added sugars drive up energy intake [9]. However, the reported differences may be overestimated since the lowest NMES consumption group may largely have be under-reporting, and also contained a higher proportion of individuals who were ‘dieting’ compared with other NMES categories. In terms of nutrient quality, a minimum amount of energy and foods may be necessary to reduce risk of deficiency.

Intakes in foods low in sugar, such as vegetables, wholemeal bread, pasta, rice, high-fibre breakfast cereal and fish, were higher in the lower-sugars consumers. Whilst it is clearly feasible to reduce NMES consumption to 5% of energy or less and still adhere to other dietary guidelines [24], it is clear that dietary choices between high and low NMES consumers are notably different, and this would involve a substantial shift in current eating habits. Changes may include consuming more protein-rich foods with fresh vegetables and high-fibre carbohydrates, including more plant foods such as beans and pulses [25], which may mean less snacking and more cooking with concomitant time and cost implications. However, in practical terms, these changes are highly challenging, and we do not have the evidence that the general public are able to do this without individual assistance.

The British Nutrition Foundation published a seven-day meal plan with suggested meals and snacks that meet the recommendations for a range of nutrients, including NMES, but this is very different from a typical diet in the UK [13]. In order for the British population to meet the new recommendations for added sugars, significant reductions in the consumption of sugar sweetened drinks, including fruit juice, beer and cider, confectionery, cakes and biscuits, would be necessary. For example, sweetened drinks could ideally be replaced with water but could also be replaced with milk or unhealthier sweetened milk-based drinks such as flavoured milk. Additional replacements for foods with both high sugar and fat, such as confectionery, cakes and biscuits, with higher-fat content foods need to be considered and avoided.

The national survey NHANES in the US found that most of the added sugars are consumed through food bought in shops such as supermarkets rather than in restaurants, so improvements in the retail sector may potentially have more impact [19], but a holistic approach is needed to target the out-of-home sector as well as supermarkets and other retailers. Diet quality in NHANES is also reduced with higher intakes of sweetened drinks and higher intakes of energy-dense foods [6]. Whether nutrient dilution effects of added sugars are counteracted by micronutrient fortification of foods is controversial. An analysis of German children and adolescents suggested that food fortification improvements to nutrient density outweighed the nutrient dilution impact of added sugars [26]. However, food fortification alone is unlikely to adequately improve diet quality at very high sugar and energy intakes.

A previous cross-sectional analysis of NDNS data reported that large portion sizes of soft drinks, were associated with higher BMI in adolescents [27]. Certainly, the evidence on the associations between specific sources of added sugars and health are strongest for sugar sweetened drinks, with recent systematic reviews of trials or cohorts in adults reporting increased weight gain [28] and increased risk of type 2 diabetes [29,30] for higher intakes of sweetened drinks. The evidence from individual food sources of added sugars is scarce, with a lack of reviews on sugar-rich foods such as confectionery, cakes and biscuits. Epidemiological evidence on different sugar substrates, such as glucose and fructose, are also scarce. A systematic review of trials on the effect of total sugar consumption on weight gain reported that higher total added sugars consumption increased the risk of weight gain [7]. However, the impact of sugars from foods was not separated from the impact from drinks [7]. Furthermore, most included trials had a short duration. There may be biological reasons why sugars in drinks are more obesogenic than other sources of sugars in foods, related to lack of satiety in energy-containing drinks [31] and glycaemic factors [32]. One suggested biological pathway is de novo lipogenesis (DNL), whereby refined carbohydrates and sugars are converted to fatty acids endogenously in the liver. Rates of DNL and fatty acid production (which leads to obesity and NAFLD) were largely increased with increased consumption of carbohydrates and sugars in parallel with decreasing levels of fat [33]. Similar findings were reported in an RCT with adolescent boys with hepatic steatosis [34].

Policies to reduce added sugars intakes were introduced by Public Health England, including a levy on sweetened drinks, reducing portion sizes of energy-dense foods and drinks, reducing promotions and marketing and encouraging reformulation [35]. Sustained behaviour change is difficult, and any one policy is unlikely to have the level of impact on dietary behaviour needed to improve population health outcomes. Contentious policies can take many years to be implemented, as seen in US attempts to reduce the sizes of drinks sold in fast food restaurants [36]. Although reductions in preference for salty foods were reported within adults [37,38,39], there is less evidence for a reduction in the preferred sweetness levels following the adoption of ‘low-sugar’ diets [40], suggesting that dietary patterns incorporating sugars-sweetened foods may potentially be more resistant to change than those incorporating salty foods. Sugar reduction policies may also need to involve programmes to change cooking practices at home in order to reduce snack foods and increase meals containing pasta, rice, and vegetables and increase the availability of healthy meals and snacks in restaurants and fast food outlets. It is also necessary to be mindful of the other equally important recommendations made to increase dietary fibre intakes, moderate total and saturated fat intakes, and select foods providing adequate amounts of vitamins and minerals.

### Strengths and Limitations

There were notable strengths and limitations in this analysis. The recommendations concerned free sugars but our current study investigated NMES, as there was limited data on free sugars from the NDNS when this study was designed. However, differences in these intakes are minor in the British diet, and many organisations, including Public Health England, have tended to present NMES and free sugars intakes interchangeably to date [41]. Information in the NDNS summary for years 7 to 8 reported that free sugars was between 15.9 and 14.1% TE for each pair of survey years, very similar to our mean NMES value of 14.9% TE [42]. Furthermore, different countries use different definitions and regions where the definition of added sugars does not include fruit juice report different results. However, our findings for foods and drinks other than fruit juice are likely to be similar. A limitation was that the NDNS data is prone to under-reporting, despite the best efforts to use data collection methods to reduce this, and is estimated to be in the region of 30% for food diaries of over 16 year-olds [14] using the Oxford and Goldberg equations. Furthermore, under-reporting may also be more common in certain groups of the population (i.e., female, or being overweight/obese) and for energy-dense foods and drinks [43,44], which had an impact on the validity of the results. We did not exclude under-reporters from the current analysis. The particularly high proportion of low-sugars consumers who reported ‘dieting’ (9%) and with implausible energy intakes suggests that this group of individuals may be dominated by individuals actively attempting to lose weight by dietary restriction, perhaps via elimination of sugar-rich foods and increased intake of protein-rich foods. Equally, it may be that this low-sugars group was dominated by individuals who were particularly poor food diary record-keepers, with both general and/or selective under-reporting of particularly sugary foods. These contributing factors made the interpretation of the results more difficult, as there were very few participants who were low-sugars consumers and reported valid energy intakes. However, reporting NMES as %TE may have negated some of the effects from under-reporters. Furthermore, the NDNS is comprised of repeated cross-sectional data. Although the NDNS is broadly representative of the dietary behaviour of the population, it is not possible to identify any causal factors. The results showing that lower sugar consumers tended to have a higher BMI were likely because these adolescents were more likely to be restricting their intakes due to excess weight. The strengths of the study were the robust methodology used to analyse the data and the use of logistic regression to generate odds ratios while adjusting for known confounders. A further strength was use of national data, which used validated dietary assessment methods.

## 5. Conclusions

The typical British adolescent diet is currently very different from the levels of free sugars recommended by the Department of Health. Low-sugars consumers have lower intakes of many sweet foods and drinks, including sugar sweetened drinks (not low calorie), fruit juice, confectionery, sugars and sweet spreads and cakes and biscuits. In addition, low-sugars consumers eat a healthier diet in terms of more vegetables and fish, and more low-fat starchy foods such as rice, pasta, and wholemeal bread. However, micronutrient intake was lower in this group than for adolescents consuming 10–15% free sugars. These findings are useful for public health nutrition policy makers in planning priorities for future action to improve the diet quality of adolescents.

## Figures and Tables

**Table 1:** Characteristics of adolescents aged 11 to 18 years in the National Diet and Nutrition Survey Y1-8 by category of non-milk extrinsic sugar consumption as a percentage of total energy after the application of Y1-8 survey weights (*n* = 2587).

Variables *	*N*	TOTAL	Quantiles of Non-Milk Extrinsic Sugars Consumption (% of Total Energy/Day)					Wald/Chi^2^ *p*-Value
			≤5	>5–10	>10–15	>15–20	>20	
No. of participants, *n* (%)	2587	-	112 (4%)	470 (17%)	795 (34%)	699 (27%)	511 (18%)	-
Age, years	2587	15 (14, 15)	15 (14, 15)	14 (14, 15)	15 (14, 15)	15 (14, 15)	15 (14, 15)	0.33
Female, %	2587	49 (46, 51)	55 (43, 67)	50 (44, 56)	51 (47, 55)	46 (41, 50)	47 (41, 52)	0.37
Body Mass Index, kg/m^2^	2493	22 (21, 22)	24 (23, 25)	22 (21, 22)	22 (21, 22)	22 (21, 22)	22 (21, 22)	0.29
Normal weight, %	1620	66 (63, 69)	51 (38, 64)	66 (59, 71)	64 (60, 69)	69 (64, 73)	69 (64, 75)	0.09
Overweight, %	349	13 (12, 15)	15 (8, 26)	12 (9, 16)	14 (11, 17)	14 (11, 18)	12 (9, 17)	
Obese, %	524	21 (18, 23)	34 (24, 47)	23 (18, 28)	22 (18, 26)	17 (14, 21)	18 (14, 23)	
Waist circumference, cm	1870	76 (75, 76)	81 (77, 85)	75 (74, 77)	76 (74, 77)	75 (74, 76)	75 (73, 77)	0.10
Waist-to-hip ratio	1869	0.81 (0.80, 0.81)	0.82 (0.80, 0.85)	0.81 (0.80, 0.82)	0.80 (0.80, 0.81)	0.81 (0.80, 0.81)	0.80 (0.79, 0.81)	0.42
Dieting	2586	7 (5, 8)	21 (13, 33)	7 (4, 10)	6 (4, 9)	7 (5, 10)	3 (2, 6)	<0.01
Achieving 5-a-day F & V	2587	8 (7, 10)	7 (3, 18)	10 (7, 15)	9 (7, 12)	8 (6, 11)	6 (4, 9)	0.33
Under-reporters †	2587	55 (52, 57)	79 (68, 88)	65 (59, 71)	54 (49, 58)	49 (44, 54)	49 (43, 54)	<0.01
Have longstanding illness	2587	16 (14, 18)	21 (12, 33)	15 (11, 21)	15 (12, 18)	19 (15, 23)	13 (9, 17)	0.18
Socio-economic status of parent								
Professional/Managerial, %	1032	42 (40, 45)	39 (27, 52)	44 (38, 50)	41 (36, 45)	45 (40, 50)	39 (34, 45)	
Intermediate, %	552	23 (21, 25)	19 (11, 30)	22 (17, 27)	24 (20, 28)	24 (20, 29)	23 (18, 28)	0.53
Routine/Manual, %	875	35 (32, 37)	42 (30, 55)	34 (29, 40)	35 (31, 40)	31 (26, 36)	38 (33, 44)	
Ethnic groups								
White, %	2309	83 (81, 85)	69 (54, 80)	79 (72, 84)	81 (77, 85)	88 (84, 92)	86 (80, 90)	<0.01
Non-white, %	276	17 (15, 19)	31 (20, 46)	21 (16, 28)	19 (15, 23)	12 (8, 16)	14 (10, 20)	
Total Energy (TE), kcal/day	2587	1761 (1734, 1788)	1390 (1258, 1523)	1593 (1542, 1644)	1778 (1735, 1821)	1830 (1777, 1884)	1872 (1800, 1944)	<0.01
Non-milk extrinsic sugars (NMES)	2587	72 (70, 74)	13 (12, 15)	34 (33, 35)	60 (58, 62)	85 (82, 88)	122 (116, 127)	<0.01
NMES, % of TE	2587	14.9 (14.5, 15.2)	3.5 (3.2, 3.8)	8.0 (7.8, 8.2)	12.6 (12.5, 12.8)	17.4 (17.2, 17.5)	24.5 (24.0, 25.1)	<0.01

* Variables expressed as mean (95% CI) for continuous variables, percentage (95% CI) for categorical variables, unless otherwise stated. † % with EI:BMR < 1.2, BMR calculated using Harris–Benedict equations.

**Table 2:** Food group intakes (g/day) of adolescents aged 11 to 18 years in the National Diet and Nutrition Survey Y1-8 by category of non-milk extrinsic sugar (NMES) consumption as a percentage of total energy after the application of Y1-8 survey weights, expressed as mean (99% CI).

Variables	Total	Consumers	Quantiles of Non-Milk Extrinsic Sugars Consumption (% of Total Energy/Day)				
		*n* (%)	≤5	>5–10	>10–15	>15–20	>20
No. of participants, *n* (%)	2587 (100%)		112 (4%)	470 (17%)	795 (34%)	699 (27%)	511 (18%)
**Food Groups**							
**Carbohydrate rich foods**							
Pasta, rice and other cereals ***	104 (98, 110)	2265 (90%)	130 (93, 167)	119 (103, 135)	107 (96 117)	99 (87, 110)	88 (75, 101)
White bread	56 (53, 59)	2274 (87%)	44 (29, 58)	58 (50, 65)	56 (50, 62)	60 (54, 66)	52 (45, 58)
Wholemeal, brown, granary, wheatgerm bread ***	21 (19, 23)	1153 (46%)	34 (19,49)	26 (19, 33)	25 (20, 29)	16 (13, 20)	14 (10, 18)
High fibre breakfast cereals **	13 (12, 15)	963 (37%)	20 (7, 34)	14 (9, 19)	16 (12, 19)	12 (9, 15)	9 (6, 12)
Other breakfast cereals	10 (9, 11)	1146 (46%)	8 (3, 12)	10 (7, 12)	10 (8, 12)	11 (9, 13)	11 (8, 14)
Biscuits ***	17 (16, 19)	1697 (67%)	7 (4, 11)	15 (12, 18)	17 (15, 20)	19 (17, 22)	20 (14, 26)
Buns, cakes, pastries and fruit pies ***	20 (18, 22)	1352 (53%)	3 (1, 4)	12 (9, 14)	21 (17, 23)	23 (19, 27)	25 (19, 31)
Puddings	11 (9, 13)	579 (24%)	7 (1, 12)	8 (4, 12)	11 (8, 14)	12 (9, 15)	11 (7, 16)
**Dairy products**							
Milk	132 (122, 142)	2137 (81%)	147 (57, 237)	141 (114, 167)	138 (120, 155)	131 (111, 151)	112 (92, 132)
Cheese ***	11 (10, 12)	1486 (60%)	8 (5, 12)	13 (10, 15)	13 (11, 14)	10 (8, 12)	8 (6, 10)
Yogurt, fromage frais and other dairy desserts **	20 (18, 22)	933 (37%)	10 (3, 17)	19 (14, 25)	22 (17, 27)	22 (18, 26)	17 (13, 22)
Ice cream ***	9 (7, 10)	698 (26%)	2 (−1, 5)	5 (3, 7)	9 (6, 12)	10 (8, 13)	11 (8, 14)
Egg and egg dishes	12 (11, 15)	896 (36%)	21 (6, 37)	14 (10, 18)	14 (10, 17)	10 (8, 13)	12 (8, 15)
Total fat spreads	8 (7, 8)	2103 (80%)	9 (6, 11)	8 (7, 9)	8 (7, 9)	8 (7, 9)	7 (5, 8)
**Potato and potato products**							
Chips, fried roast potatoes and potato dishes	50 (47, 53)	2022 (78%)	42 (22, 61)	43 (36, 50)	53 (47, 59)	52 (47, 57)	51 (44, 59)
Other potatoes, potato salads and dishes	31 (28, 34)	1474 (54%)	34 (17, 52)	32 (25, 38)	34 (29, 39)	31 (26, 35)	25 (20, 30)
Crisps and savoury snacks **	13 (12, 13)	1775 (69%)	10 (4, 15)	11 (9, 13)	12 (10, 13)	14 (12, 16)	13 (11, 16)
**Sugar, preserves and confectionery**							
Sugars, preservatives and sweet spreads ***	7 (6, 7)	1553 (61%)	2 (0, 3)	4 (3, 5)	6 (5, 8)	8 (7, 10)	9 (7, 11)
Sugar confectionery ***	7 (6, 8)	848 (33%)	1 (0, 1)	2 (1, 3)	4 (3, 5)	8 (6, 10)	15 (12, 19)
Chocolate confectionery ***	12 (11, 13)	1498 (56%)	3 (1, 4)	6 (5, 8)	10 (9, 12)	15 (12, 17)	18 (14, 21)
**Beverages**							
Fruit juice ***	78 (69, 87)	1218 (49%)	7 (1, 13)	31 (23, 39)	68 (57, 80)	99 (82, 117)	125 (91, 160)
Soft drinks, not low calorie ***	230 (213, 247)	1957 (75%)	12 (2, 21)	65 (52, 78)	164 (143, 185)	280 (251, 308)	484 (432, 535)
Soft drinks, low calorie **	184 (165, 202)	1454 (54%)	183 (71, 294)	225 (179, 271)	199 (162, 237)	160 (131, 189)	151 (114, 189)
Beer, lager, cider and perry ***	31 (19, 42)	2408 (93%)	4 (−3, 11)	16 (−1, 34)	30 (13, 47)	29 (10, 49)	52 (18, 98)
**Disaggregated Food Groups †**							
Total Fruit	59 (54, 63)	2169 (83%)	53 (30, 75)	69 (54, 83)	61 (53, 69)	55 (47, 63)	51 (40, 62)
Total vegetables ***	112 (107, 117)	2570 (95%)	115 (92, 138)	119 (107, 138)	121 (113, 130)	110 (101, 119)	89 (80, 98)
Total meat	97 (93, 101)	2500 (96%)	99 (72, 125)	97 (87, 106)	99 (92, 106)	98 (91, 105)	91 (83, 99)
Total fish ***	12 (11, 14)	1214 (50%)	18 (10, 25)	16 (10, 22)	12 (10, 15)	11 (9, 13)	8 (6, 11)

*** = *p* < 0.001, ** = *p* < 0.01, significant difference across NMES groups using wald test. † Disaggregated food groups include estimated portions of foods that are in composite dishes in order to provide a more complete estimate of intake at the individual food level.

**Table 3:** Daily mean (99% CI) nutrient intakes of adolescents aged 11 to 18 years in the National Diet and Nutrition Survey Y1-8 by percentage of non-milk extrinsic sugars consumption of total energy (*n* = 2587) after the application of survey weights.

Variables	Total	Quantiles of Non-Milk Extrinsic Sugars Consumption (% of Total Energy/Day)					*p*-Value
		≤5	>5–10	>10–15	>15–20	>20	
No. of participants, *n*	2587 (100%)	112 (4%)	470 (17%)	795 (34%)	699 (27%)	511 (18%)	
**Macronutrients**							
Total Energy (TE), kcal/day	1761 (1726, 1797)	1390 (1216, 1565)	1593 (1526, 1661)	1778 (1721, 1835)	1830 (1760, 1901)	1872 (1777, 1967)	<0.01
Food Energy (FE), kcal/day	1750 (1714, 1785)	1389 (1215, 1563)	1585 (1519, 1652)	1765 (1709, 1822)	1820 (1750, 1890)	1855 (1761, 1949)	<0.01
Total Energy (TE), kJ/day	7417 (7268, 7569)	5854 (5120, 6588)	6707 (6425, 6988)	7485 (7247, 7723)	7708 (7412, 8002)	7890 (7490, 8290)	<0.01
Food Energy (FE), kJ/day	7369 (7221, 7516)	5847 (5114, 6581)	6672 (6393, 6950)	7432 (7195, 7668)	7664 (7370, 7958)	7821 (7426, 8216)	<0.01
Protein (g)	66 (64, 67)	66 (54, 78)	68 (64, 71)	68 (66, 71)	65 (63, 68)	60 (57, 63)	<0.01
Fat, % of TE	34 (33, 34)	35 (33, 37)	35 (34, 36)	35 (34, 35)	33 (33, 34)	31 (30, 32)	<0.01
CHO, % of TE	51 (50, 51)	46 (44, 48)	48 (47, 48)	49 (49, 50)	52 (51, 52)	56 (55, 56)	<0.01
Total sugars	101 (98, 104)	38 (32 45)	64 (61, 68)	91 (87, 94)	114 (111, 119)	149 (140, 156)	<0.01
Total sugars, % of TE	21 (21, 22)	10 (9, 12)	15 (15 16)	19 (19, 20)	24 (23, 24)	30 (29, 31)	<0.01
Non-milk extrinsic sugars (NMES)	72 (69, 74)	13 (11, 15)	34 (32, 35)	60 (58, 62)	85 (81, 88)	122 (115, 128)	<0.01
NMES, % of TE	15 (15, 15)	4 (3, 4)	8 (8, 8)	13 (12, 13)	17 (17, 18)	25 (24, 25)	<0.01
AOAC fibre (g)	16 (15, 16)	15 (13, 17)	16 (15, 17)	17 (16, 17)	16 (15, 16)	14 (13, 15)	<0.01
Non-starch polysaccharides (NSP) (g)	12 (12, 12)	11 (10, 13)	12 (11, 13)	12 (12, 13)	12 (11, 12)	10 (9, 11)	<0.01
Alcohol (g)	1.7 (1.2, 2.1)	0.2 (−0.1, 0.5)	1.2 (0.2, 2.0)	1.8 (0.8, 2.8)	1.5 (0.7, 2.2)	2.4 (0.8, 3.9)	<0.01
**Micronutrients**							
Vitamin A, µg/day	624 (590, 680)	495 (376, 614)	653 (548, 759)	634 (588, 682)	624 (557, 690)	610 (537, 683)	0.07
Thiamin, mg/day	1.4 (1.4, 1.4)	1.3 (1.1, 1.5)	1.4 (1.3, 1.4)	1.5 (1.4, 1.5)	1.4 (1.4, 1.5)	1.3 (1.3, 1.4)	0.01
Riboflavin, mg/day	1.4 (1.4, 1.5)	1.3 (0.9, 1.6)	1.4 (1.3, 1.5)	1.5 (1.4, 1.5)	1.4 (1.4, 1.5)	1.4 (1.3, 1.5)	0.30
Niacin equivalents, mg/day	32 (31, 33)	31 (26, 36)	32 (30, 34)	33 (32, 34)	32 (30, 33)	31 (29, 33)	0.40
Vitamin B6, mg/day	1.9 (1.8, 1.9)	1.6 (1.3, 1.9)	1.7 (1.6, 1.9)	1.8 (1.7, 1.9)	1.9 (1.8, 2.0)	2.1 (1.8, 2.4)	<0.01
Vitamin B12, µg/day	4.2 (4.0, 4.4)	4.0 (3.0, 5.0)	4.4 (4.0, 4.7)	4.3 (4.0, 4.6)	4.1 (3.8, 4.4)	4.1 (3.7, 4.5)	0.37
Folate, µg/day	205 (200, 211)	181 (153, 209)	201 (186, 216)	214 (196, 220)	208 (197, 220)	194 (181, 206)	<0.01
Vitamin C, mg/day	80 (76, 83)	51 (40, 63)	61 (55, 68)	78 (72, 84)	84 (78, 90)	99 (88, 111)	<0.01
Vitamin D, µg/day	2.2 (2.1, 2.3)	2.1 (1.7, 2.5)	2.2 (2.1, 2.4)	2.3 (2.1, 2.5)	2.1 (2.0, 2.2)	1.9 (1.7, 2.2)	<0.01
Vitamin E, mg/day	8.8 (8.5, 9.1)	7.7 (6.8, 8.5)	8.5 (8.0, 9.0)	9.3 (8.8, 9.7)	8.9 (8.5, 9.3)	8.3 (7.7, 8.9)	<0.01
Iron, mg/day	9.5 (9.3, 9.7)	8.3 (6.9, 9.6)	9.3 (8.9, 9.7)	9.9 (9.5, 10.2)	9.6 (9.2, 10.0)	9.0 (8.4, 9.5)	<0.01
Calcium, mg/day	782 (758, 805)	664 (525, 804)	774 (722, 826)	812 (772, 853)	788 (746, 830)	750 (693, 808)	0.03
Magnesium, mg/day	210 (205, 214)	189 (162, 216)	207 (195, 219)	217 (209, 225)	211 (203, 219)	201 (190, 211)	<0.01
Potassium, mg/day	2305 (2255, 2355)	2024 (1710, 2339)	2211 (2098, 2324)	2391 (2300, 2484)	2355 (2265, 2445)	2224 (2105, 2344)	<0.01
Zinc, mg/day	7.3 (7.2, 7.5)	7.2 (6.0, 8.3)	7.7 (7.2, 8.2)	7.7 (7.4, 8.0)	7.2 (6.9, 7.5)	6.6 (6.2, 7.0)	<0.01
Iodine, mg/day	124 (119, 129)	105 (80, 130)	128 (115, 140)	130 (119, 141)	122 (114, 130)	118 (108, 129)	0.07
Sodium, mg/day	2114 (2063, 2164)	1898 (1631, 2165)	2052 (1944, 2161)	2185 (2096, 2274)	2150 (2049, 2250)	2038 (1908, 2168)	0.01

TE = total energy, FE= Food energy, AOAC = Association of Official Analytical Chemists.

**Table 4:** Proportion of adolescents aged 11 to 18 years in the National Diet and Nutrition Survey with micronutrient intakes below LRNI by percentage of non-milk extrinsic sugars consumption of total energy (*n* = 2587) after the application of survey weights, expressed as percentage (99% CI).

Variables	Total	Quantiles of Non-Milk Extrinsic Sugars Consumption (% of Total Energy/Day)					
		≤5	>5–10	>10–15	>15–20	>20	*p*-Value
No. of participants, *n*	2587 (100%)	112 (4%)	470 (17%)	795 (34%)	699 (27%)	511 (18%)	
**Micronutrients**							
Vitamin A, µg/day	16 (13, 18)	21 (12, 35)	16 (11, 24)	12 (9, 17)	18 (13, 23)	17 (12, 25)	0.09
Riboflavin, mg/day	16 (13, 18)	39 (23, 56)	20 (15, 27)	13 (9, 17)	13 (9, 17)	16 (11, 22)	<0.01
Vitamin B12, µg/day	2 (1, 3)	5 (2, 13)	1 (1, 3)	1 (0, 4)	1 (0, 3)	2 (1, 6)	0.31
Folate, µg/day	7 (5, 9)	12 (5, 26)	6 (3, 11)	5 (3, 8)	6 (4, 10)	12 (7, 18)	0.03
Vitamin C, mg/day	1 (1, 2)	4 (1, 19)	2 (1, 6)	0 (0, 2)	1 (0, 5)	2 (1, 5)	0.11
Iron, mg/day	28 (25, 31)	44 (29, 61)	30 (23, 37)	25 (20, 30)	25 (20, 31)	33 (26, 41)	0.01
Calcium, mg/day	15 (13, 17)	31 (18, 49)	18 (13, 25)	10 (7, 14)	13 (10, 18)	18 (13, 25)	<0.01
Magnesium, mg/day	39 (36, 42)	51 (35, 67)	45 (37, 52)	33 (28, 38)	37 (31, 43)	44 (37, 52)	<0.01
Potassium, mg/day	25 (22, 28)	37 (23, 55)	30 (23, 38)	22 (17, 27)	21 (17, 27)	29 (22, 36)	0.01
Zinc, mg/day	19 (16, 21)	27 (15, 44)	19 (13, 26)	13 (10, 17)	19 (14, 25)	27 (21, 35)	<0.01
Iodine, mg/day	18 (16, 21)	37 (22, 54)	21 (15, 28)	14 (10, 18)	16 (12, 21)	22 (16, 28)	<0.01

## Supplementary Materials

The following are available online at https://www.mdpi.com/2072-6643/11/7/1621/s1, Table S1: Categories of food group, respective portion sizes and foods included, Table S2: The age and gender-adjusted odds (99% confidence interval, *p*-value) of being a low NMES consumer (≤5% total energy) in all adolescents aged 11 to 18 years with increasing food intake by typical portions (g/day), and after excluding participants who reported being a diet or on a special diet (*n* = 152) in the NDNS with increasing consumption of various foods by portion (g/day), Table S3: Percentage (99% CI) of adolescents aged 11 to 18 years in the National Diet and Nutrition Survey with micronutrient intakes below LRNI by percentage of non-milk extrinsic sugars consumption of total energy excluding dieters (*n* = 2434) after the application of survey weights, Figure S1: Percentage of adolescents aged 11 to 18 years excluding dieters in the National Diet and Nutrition Survey with micronutrient intakes below LRNI by percentage of non-milk extrinsic sugars consumption of total energy (*n* = 2434) after the application of survey weights.
