# Supplementary material for: Non-Milk Extrinsic Sugars Intake and Food and Nutrient Consumption Patterns among Adolescents in the UK National Diet and Nutrition Survey, Years 2008–16
Source: Nutrients. 2019 Jul 17;11(7):1621. doi: 10.3390/nu11071621 (PMC6682974; doi:10.3390/nu11071621)

Supplementary Materials

Table S1. Categories of food group, respective portion sizes and foods included.

Category	Food group (portion size)	Foods included
Carbohydrate-rich foods	Pasta, rice and other miscellaneous cereals (100 g/day)	Pizza<br>Pasta (manufactured, ready meals, homemade, others)<br>Rice (manufactured, ready meals, homemade, others)<br>Other cereals
	White bread (25 g/day)	White bread (not high fibre, not multiseed)
	Wholemeal and brown bread (25 g/day)	Wholemeal bread<br>Brown bread<br>Granary bread<br>Wheatgerm bread
	High fibre breakfast cereals (25 g/day)	High fibre breakfast cereals
	Other breakfast cereals (25 g/day)	Other breakfast cereals (not high fibre)
	Biscuits (15 g/day)	Biscuits (manufactured/retail)<br>Biscuits (homemade)
	Buns, cakes, pastries and fruit pies (50 g/day)	Fruit pies (manufactured)<br>Fruit pies (homemade)<br>Buns, cake and pastries (manufactured)<br>Buns, cake and pastries (homemade)
	Puddings (50 g/day)	Cereal based milk puddings (manufactured)<br>Cereal based milk puddings (homemade)<br>Sponge puddings (manufactured)<br>Sponge puddings (homemade)<br>Other cereal based puddings (manufactured)<br>Other cereal based puddings (homemade)
Dairy products	Total milk (100 g/day)	Whole milk<br>Semi-skimmed milk<br>1% milk<br>Skimmed milk
	Yogurt, fromage frais and other dairy desserts (150 g/day)	Yogurt<br>Fromage frais and other dairy desserts (manufactured)<br>Dairy desserts (homemade)
	Ice cream (25 g/day)	Ice cream
Other solid foods	Eggs and egg dishes (50 g/day)	Manufactured egg products including ready meals<br>Other eggs and egg dishes including homemade
	Total fat spread (10 g/day)	Butter<br>Polyunsaturated margarine<br>Polyunsaturated oils<br>Polyunsaturated low fat spread<br>Low fat spread not polyunsaturated<br>Block margarine<br>Soft margarine not polyunsaturated<br>Other cooking fats and oils not polyunsaturated<br>Reduced fat spread (polyunsaturated)<br>Reduced fat spread (not polyunsaturated)
	Chips, fried and roast potatoes and potato products (100 g/day)	Chips purchased including takeaway<br>Other manufactured potato products fried/baked<br>Other fried/roast potatoes (including homemade dishes)
	Other potatoes, potato salads and dishes (100 g/day)	Other potato products and dishes (manufactured)<br>Other potatoes (including homemade dishes)
	Crisps and savoury snacks (20 g/day)	Crisps and savoury snacks

Sweets and confectionery	Sugars, preserves and sweet spreads (20 g/day)	Sugar<br>Preserves<br>Sweet spreads, fillings and icing
	Sugar confectionery (10 g/day)	Sugar confectionery
	Chocolate confectionery (10 g/day)	Chocolate confectionery
Alcoholic and non-alcoholic beverages	Fruit juice	Fruit juice<br>Smoothies
	Soft drinks, not diet (100 g/day)	Soft drinks not low calorie, concentrated<br>Soft drinks not low calorie, carbonated<br>Soft drinks not low calorie, ready to drink, still
	Soft drinks, diet (100 g/day)	Soft drinks low calorie, concentrated<br>Soft drinks low calorie, carbonated<br>Soft drinks low calorie, ready to drink, still
	Wine (100 g/day)	Wine<br>Fortified wine<br>Low alcohol and alcohol free wine
	Beer, lager, cider and perry	Beers and lagers<br>Low alcohol and alcohol free beer and lager<br>Cider and perry<br>Low alcohol and alcohol free cider and perry<br>Alcoholic soft drinks (Alcopops)
Disaggregated food groups	Total fruit (80 g/day)	Total fruit
	Total vegetable (80 g/day)	Total vegetable
	Total meat (100 g/day)	Total meat
	Total fish (50 g/day)	Total fish

Table S2. The age and gender-adjusted odds (99% confidence interval, p-value) of being a low NMES consumer (≤5% total energy) in adolescents aged 11 to 18 years with increasing food intake by typical portions (g/day), and after excluding participants who reported being a diet or on a special diet (n=152) in the National Diet and Nutrition Survey with increasing consumption of various foods by portion (g/day)

	**Odds of being categorised as ≤5% NMES of total energy (99% CI, p-value)**			
**FOOD GROUP**	Unadjusted	Age, gender-adjusted odds		
	All participants	All participants	All excluding dieters	also adjusting for dieters
**Carbohydrate-rich foods**				
Pasta, rice and other cereals, 100 g/day	1.32 (0.95, 1.83), 0.03	1.33 (0.95, 1.85), 0.03	1.23 (0.83, 1.85), 0.17	1.36 (0.97, 1.91), 0.02
White bread, g/day, 25 g/day	0.84 (0.67, 1.06), 0.06	0.85 (0.67, 1.07), 0.08	0.87 (0.67, 1.11), 0.15	0.86 (0.68, 1.10), 0.11
Wholemeal and brown bread, 25 g/day	1.24 (1.03, 1.49), <0.01	1.27 (1.05, 1.53), <0.01	1.21 (0.98, 1.49), 0.02	1.25 (1.04, 1.50), <0.01
High fibre breakfast cereals, 25 g/day	1.14 (0.94, 1.38), 0.08	1.14 (0.97, 1.37), 0.05	1.15 (0.95, 1.40), 0.06	1.15 (0.95, 1.39), 0.05
Other breakfast cereals, 25 g/day	0.71 (0.38, 1.32), 0.15	0.74 (0.39, 1.41), 0.24	0.83 (0.43, 1.61), 0.47	0.75 (0.39, 1.45), 0.26
Biscuits, 15 g/day	0.51 (0.33, 0.79), <0.01	0.52 (0.33, 0.82), <0.01	0.49 (0.30, 0.81), <0.01	0.53 (0.33, 0.83), <0.01
Buns, cakes, pastries and fruit pies, 50 g/day	0.02 (0.00, 0.12), <0.01	0.02 (0.00, 0.13), <0.01	0.03 (0.00, 0.19), <0.01	0.02 (0.00, 0.14), <0.01
Puddings, 50 g/day	0.68 (0.32, 1.41), 0.18	0.70 (0.35, 1.43), 0.20	0.48 (0.17, 1.37), 0.07	0.72 (0.35, 1.48), 0.23
**Dairy products**				
Milk, 100 g/day	1.05 (0.82, 1.35), 0.60	1.08 (0.85, 1.37), 0.41	1.13 (0.93, 1.37), 0.11	1.09 (0.86, 1.38), 0.34
Cheese, 25g/day	0.68 (0.37, 1.26), 0.11	0.68 (0.37, 1.26), 0.11	0.68 (0.37, 1.25), 0.10	0.72 (0.38, 1.33), 0.16
Yogurt, fromage frais, other dairy desserts , 150 g/day	0.17 (0.02, 1.17), 0.02	0.17 (0.02, 1.25), 0.02	0.11 (0.01, 1.33), 0.02	0.16 (0.02, 1.26), 0.02
Ice cream, 25 g/day	0.27 (0.05, 1.35), 0.04	0.28 (0.06, 1.35), 0.04	0.06 (0.01, 0.45), <0.01	0.29 (0.06, 1.33), 0.04
**Other solid foods**				
Egg and egg dishes, 50 g/day	1.62 (0.95, 2.75), 0.02	1.63 (0.96, 2.79), 0.02	1.79 (1.05, 3.04), <0.01	1.69 (0.97, 2.93), 0.02
Total fat spreads, 10 g/day	1.11 (0.81, 1.54), 0.39	1.13 (0.81, 1.58), 0.33	1.02 (0.70, 1.47), 0.92	1.17 (0.84, 1.64), 0.21
Chips, fried roast potatoes and potato dishes, 100 g/day	0.63 (0.19, 2.08), 0.32	0.62 (0.18, 2.09), 0.31	0.58 (0.13, 2.55), 0.34	0.63 (0.19, 2.08), 0.32
Other potatoes, potato salads and dishes, 100 g/day	1.23 (0.45, 3.32), 0.59	1.24 (0.47, 3.29), 0.57	1.58 (0.57, 4.32), 0.24	1.30 (0.49, 3.44), 0.50
Crisps and savoury snacks, 20 g/day	0.74 (0.34, 1.58), 0.30	0.75 (0.35, 1.60), 0.33	0.84 (0.39, 1.84), 0.58	0.78 (0.37, 1.67), 0.40
**Sweets and confectionary**				
Sugars, preserves and sweet spreads, 10 g/day	0.03 (0.00, 0.38), 0.01	0.03 (0.00, 0.37), <0.01	0.04 (0.00, 0.59), <0.01	0.03 (0.00, 0.38), <0.01
Sugar confectionery, 10 g/day	0.18 (0.06, 0.54), <0.01	0.18 (0.06, 0.56), <0.01	0.18 (0.05, 0.61), <0.01	0.19 (0.06, 0.57), <0.01
Chocolate confectionery, 10 g/day	0.37 (0.19, 0.71), <0.01	0.37 (0.19, 0.72), <0.01	0.39 (0.19, 0.80), <0.01	0.38 (0.19, 0.73), <0.01
**Alcoholic and non-alcoholic beverages**				
Fruit juice, 125 g/day	0.06 (0.01, 0.27), <0.01	0.06 (0.01, 0.27), <0.01	0.05 (0.01, 0.30), <0.01	0.06 (0.01, 0.27), <0.01
Soft drinks, not low calorie, 100 g/day	0.11 (0.04, 0.27), <0.01	0.11 (0.04, 0.28), <0.01	0.12 (0.05, 0.33), <0.01	0.11 (0.04 0.28), <0.01

Soft drinks, low calorie, 100 g/day	1.00 (0.86, 1.15), 0.98	1.00 (0.87, 1.16), 0.98	0.99 (0.82, 1.19), 0.91	0.99 (0.85, 1.14), 0.80
Beer, lager, cider and perry, 100 g/day	0.64 (0.30, 1.37), 0.13	0.58 (0.24, 1.42), 0.12	0.41 (0.09, 1.97), 0.14	0.57 (0.22, 1.47), 0.13
**Disaggregated food groups**				
Total fruit, 80 g/day	0.90 (0.60, 1.37), 0.54	0.92 (0.60, 1.40), 0.60	0.81 (0.51, 1.28), 0.23	0.92 (0.60, 1.41), 0.62
Total vegetables, 80 g/day	1.05 (0.74, 1.50), 0.72	1.04 (0.73, 1.46), 0.79	1.15 (0.80, 1.64), 0.34	1.04 (0.73, 1.49), 0.79
Total meat, 100 g/day	1.07 (0.49, 2.35), 0.82	1.12 (0.50, 2.50), 0.72	1.21 (0.46, 3.18), 0.60	1.12 (0.50, 2.54), 0.71
Total fish, 50 g/day	1.54 (0.91, 2.60), 0.03	1.54 (0.92, 2.58), 0.03	1.63 (0.94, 2.84), 0.02	1.58 (0.93, 2.66), 0.03

Table S3. Percentage (99% CI) of adolescents aged 11 to 18 years in the National Diet and Nutrition Survey with micronutrient intakes below LRNI by percentage of non-milk extrinsic sugars consumption of total energy excluding dieters (n = 2434) after the application of survey weights.

VARIABLES	TOTAL	Quantiles of non-milk extrinsic sugars consumption (% of total energy/day)					
		≤5	>5-10	>10 – 15	>15 – 20	>20	p-value
No. of participants, n	2434 (100%)	94 (4%)	439 (18%)	748 (31%)	661 (27%)	492 (20%)	
MICRONUTRIENTS							
Vitamin A, %	15 (13, 18)	22 (12, 36)	15 (10, 22)	11 (8, 15)	17 (13, 23)	17 (12, 25)	0.03
Riboflavin, %	15 (13, 18)	37 (20, 57)	19 (13, 26)	13 (9, 18)	13 (9, 18)	15 (11, 22)	<0.01
Vitamin B12, %	2 (1, 3)	5 (2, 16)	1 (0, 3)	1 (0, 5)	1 (0, 4)	2 (1, 6)	0.34
Folate, %	7 (5, 9)	15 (6, 31)	5 (3, 10)	5 (3, 9)	6 (3, 10)	11 (7, 18)	0.03
Vitamin C, %	1 (1, 2)	5 (1, 23)	2 (1, 7)	0 (0, 2)	1 (0, 5)	2 (1, 5)	0.09
Iron, %	27 (24, 30)	41 (24, 60)	28 (21, 36)	24 (19, 29)	25 (20, 31)	32 (25, 40)	0.06
Calcium, %	14 (12, 17)	31 (16, 51)	16 (11, 24)	11 (7, 15)	13 (9, 18)	17 (12, 23)	<0.01
Magnesium, %	38 (35, 41)	50 (32, 68)	43 (35, 51)	32 (27, 38)	37 (30, 43)	43 (36, 51)	<0.01
Potassium, %	24 (22, 27)	35 (20, 55)	29 (21, 37)	21 (17, 28)	22 (17, 28)	28 (21, 35)	0.06
Zinc, %	18 (16, 21)	28 (14, 49)	18 (12, 25)	12 (9, 17)	19 (14, 25)	27 (20, 34)	<0.01
Iodine, %	17 (15, 20)	31 (16, 51)	20 (14, 28)	14 (11, 19)	16 (12, 21)	21 (15, 27)	0.04

Figure S1. Percentage of adolescents aged 11 to 18 years excluding dieters in the National Diet and Nutrition Survey with micronutrient intakes below LRNI by percentage of non-milk extrinsic sugars consumption of total energy (n = 2434) after the application of survey weights.

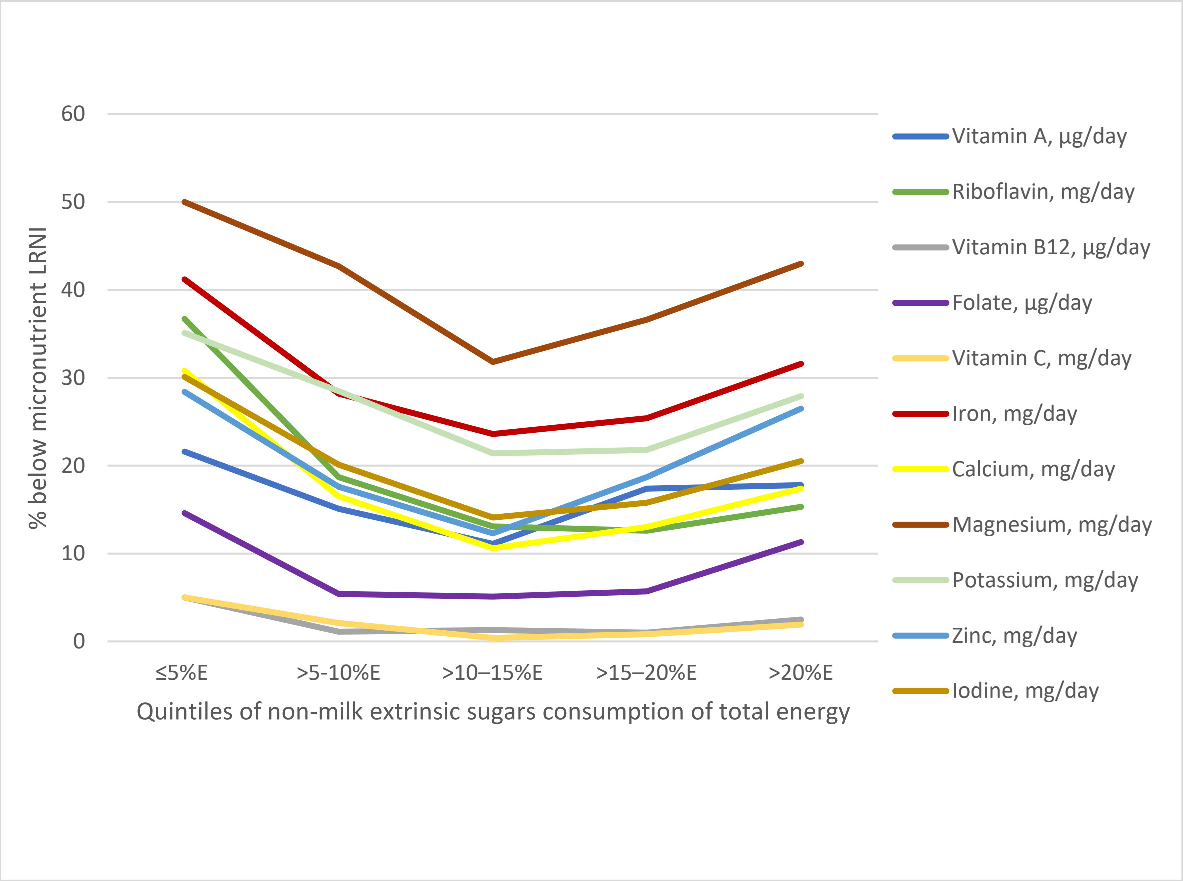

Supplement: Supplementary file 1 [file nutrients-11-01621-s001.pdf]
